# Prognostic value of the neutrophil percentage-to-albumin ratio in patients with IgA nephropathy

**DOI:** 10.1186/s12882-026-05235-y

**Published:** 2026-07-29

**Authors:** Shuo Li, Yan Huang, Meiran Cao, Jingfu Wang

**Affiliations:** Department of Nephrology, Affiliated Hospital of Chengde Medical University, Chengde, Hebei Province China

**Keywords:** IgA nephropathy, Neutrophil percentage-to-albumin ratio, End-stage renal disease, Prognosis, Inflammation

## Abstract

The neutrophil percentage-to-albumin ratio (NPAR) has emerged as a biomarker for systemic inflammation; however, its association with the prognosis of IgA nephropathy (IgAN) remains unclear. In this retrospective cohort study, we aimed to evaluate the relationship between NPAR and IgAN and enrolled 389 patients with biopsy-proven primary IgAN treated at the Affiliated Hospital of Chengde Medical University between January 2013 and July 2024. Baseline demographic, clinical, and pathological data were collected. The NPAR was calculated as neutrophil percentage (NEUT%) × 100 / albumin (g/dL). The optimal cut-off value of the NPAR for predicting end-stage renal disease (ESRD) was determined using receiver operating characteristic (ROC) curve analysis; the patients were divided into high and low NPAR groups accordingly. The ROC curve showed that the optimal cut-off value of the NPAR for predicting ESRD was 18.54, with an area under the curve of 0.670 (sensitivity 55.3%, specificity 74.6%). Patients in the high NPAR group were significantly older and had a higher prevalence of anemia, as well as higher NEUT%, 24-h UPE, and incidence of ESRD, whereas eGFR and albumin levels were significantly lower (all *P* < 0.05); pathologically, they were more prone to endocapillary hypercellularity (E1) and tubular atrophy/interstitial fibrosis (T1-2) (*P* < 0.05). Correlation analysis showed that the NPAR was positively correlated with age, anemia, 24-hour urine protein, E lesion, and T lesion, and negatively correlated with eGFR (all *P* < 0.05). Multivariate Cox regression analysis demonstrated that an NPAR ≥ 18.54 was an independent risk factor for progression to ESRD in IgAN patients (HR = 2.176, 95%CI: 1.058～4.472, *P* = 0.034). Subgroup analysis showed that NPAR demonstrated a consistent prognostic discriminative trend across most subgroups, whereas no significant predictive effect was observed in the M0 subtype. Restricted cubic splines (RCS) suggested a linear positive correlation between NPAR and ESRD risk (*P* for non-linearity = 0.5569). An elevated NPAR is independently associated with severe clinicopathological features and poor renal prognosis in patients with IgAN. As a convenient and cost-effective inflammatory marker, given its limited discriminative ability (AUC = 0.670), NPAR can only serve as an auxiliary reference in clinical assessment, and its practical value requires further external validation.

**Supplementary Information:**

The online version contains supplementary material available at https://doi.org/10.1186/s12882-026-05235-y.

## Introduction

IgA nephropathy (IgAN) is a primary glomerulonephritis characterized pathologically by predominant IgA deposition in the glomerular mesangium, followed by mesangial cell proliferation and matrix expansion. It ranks first among the primary glomerulonephritides worldwide; IgAN is also an important cause of end-stage renal disease (ESRD) [1]. The clinical presentation of IgAN is markedly heterogeneous, encompassing a broad spectrum that ranges from asymptomatic microscopic hematuria to nephrotic-range proteinuria, hypertension, and progressive renal failure culminating in ESRD. Its incidence is high, with an estimated global incidence rate of 2.5 cases per 100,000 individuals [2]. The prognosis is relatively poor, making it a common cause of ESRD; approximately 36% of patients gradually lose renal function and progress to ESRD within 20 years of diagnosis [3]. Even patients with clinically early-stage IgAN still face the risk of renal insufficiency. This situation not only significantly diminishes patients’ quality of life but also imposes a substantial economic burden. Therefore, early identification and active intervention for the risk factors influencing the prognosis of IgAN are particularly important.

To date, the pathogenesis of IgAN is not yet fully understood. Studies suggest that the pathogenesis of IgAN may be associated with immune-inflammatory responses [4]. In recent years, the neutrophil percentage-to-albumin ratio (NPAR), an emerging biomarker reflecting both inflammatory levels and nutritional status, has been found to be associated with adverse clinical outcomes in various diseases, such as cardiovascular and cerebrovascular diseases [5, 6], tumors [7], respiratory diseases [8], and autoimmune diseases [9]. Furthermore, Wang et al. investigated the association between the NPAR and all-cause mortality in patients with acute kidney injury (AKI). In multivariate analysis, they found that, after adjusting for sex, age, race, and other confounders, an elevated NPAR in patients with AKI was associated with an increased risk of all-cause mortality [10]. Rao et al. also reported that an elevated NPAR could predict all-cause and cardiovascular mortality in patients with advanced chronic kidney disease (CKD), suggesting that individuals with higher NPAR levels have an increased risk of death and significantly reduced survival rates in the context of CKD [11]. Similarly, the NPAR has demonstrated good predictive value in assessing the prognosis of patients undergoing hemodialysis and peritoneal dialysis [12, 13]. However, whether the NPAR is associated with the prognosis of patients with IgAN has not been reported to date. Accordingly, we aimed to investigate the impact of the NPAR on IgAN and to explore its value in evaluating the prognosis of patients with IgAN.

## Materials and methods

### Study population

**Fig. 1:**
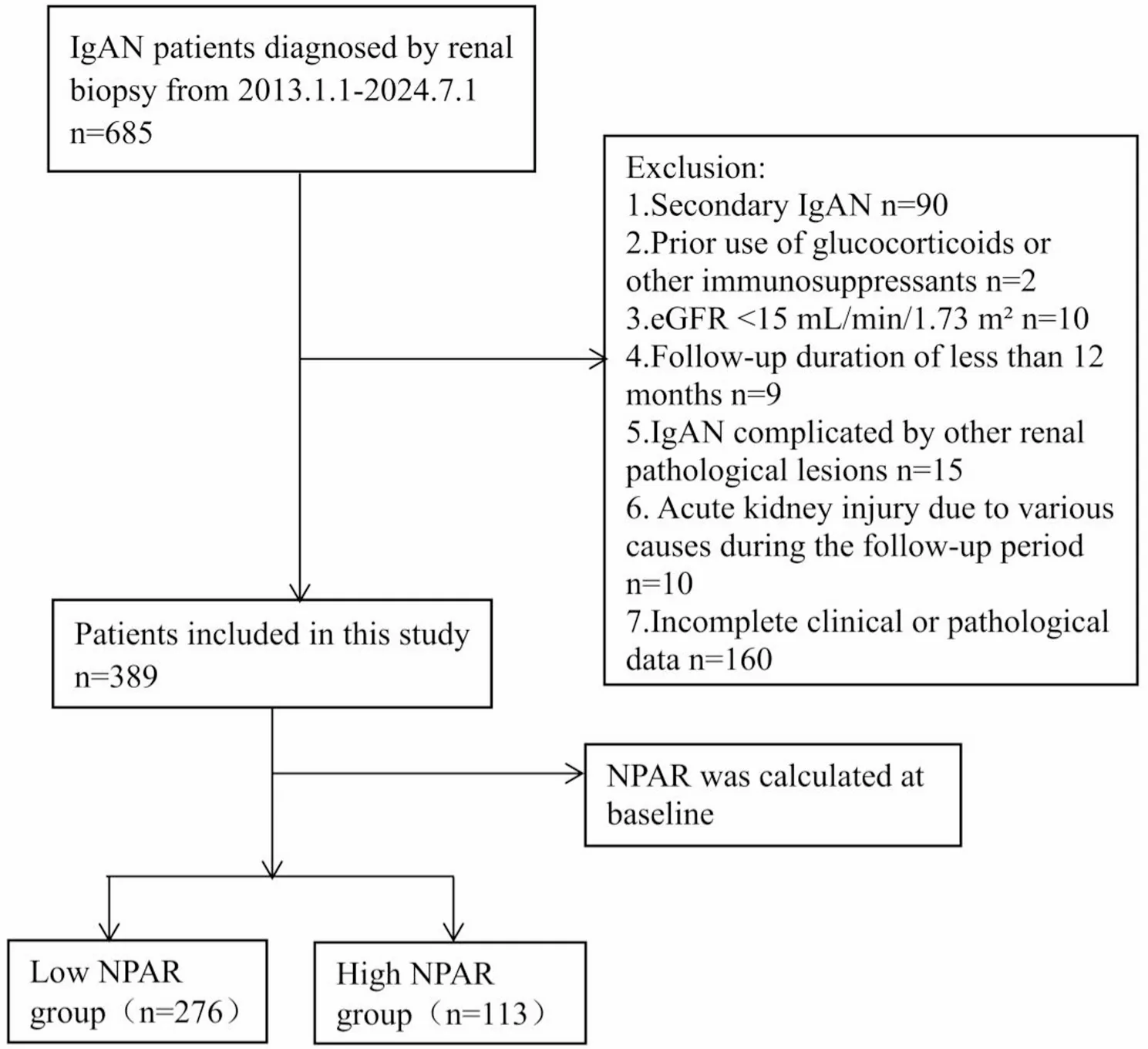
The flow chart of the study

We retrospectively enrolled treatment-naïve patients with primary IgAN who were diagnosed by renal biopsy at the Department of Nephrology, Affiliated Hospital of Chengde Medical University from January 2013 to July 2024. Their clinical and renal biopsy pathological data were collected. The exclusion criteria were: (1) Secondary IgAN (e.g., due to systemic lupus erythematosus, liver cirrhosis, Henoch–Schönlein purpura); (2) Prior use of corticosteroids or other immunosuppressants; (3) eGFR < 15 ml/min/1.73 m² at the time of renal biopsy; (4) Follow-up duration of less than 12 months; (5) IgAN complicated by other renal pathological lesions, such as membranous nephropathy; (6) Acute kidney injury due to various causes during the follow-up period; (7) Incomplete clinical or pathological data. Ultimately, 389 patients with IgAN who met the criteria were included in the study. This study was approved by the Ethics Committee of the Affiliated Hospital of Chengde Medical University (Approval No. CYFYLL2023409), and all research procedures strictly adhered to the principles of the Declaration of Helsinki. All patients provided informed consent (Fig. 1).

### Clinical data

In this study, we meticulously reviewed the medical records of each patient through the electronic medical record system and collected baseline demographic and laboratory data at the time of renal biopsy. This included age, sex, history of hypertension, hemoglobin (HB), neutrophil percentage (NEUT%), platelet count (PLT), albumin (ALB), 24-hour urine protein excretion (24-h UPE), estimated glomerular filtration rate (eGFR), and treatment regimens. The NPAR was calculated using the formula: neutrophil percentage (%) × 100 / albumin (g/dL). The neutrophil percentage was chosen over the absolute neutrophil count because it reflects the relative proportion of neutrophils, corrects for fluctuations in total white blood cell count caused by non-inflammatory factors, and is a directly reported parameter with a stable reference range. This definition has been widely used in previous studies [14, 15]. Hypertension was defined as blood pressure > 140/90 mmHg or current use of antihypertensive medication. eGFR was calculated using the CKD-EPI formula. Anemia was defined as hemoglobin concentration < 120 g/L in men and < 110 g/L in women. Hyperuricemia was defined as serum uric acid level > 420 µmol/L in men and > 360 µmol/L in women.

### Pathological data

Renal histopathological classification was determined according to the MEST-C scoring system [16] based on the Oxford classification. Pathological features included the following: (1) Mesangial hypercellularity (M): M0, < 4 mesangial cells/mesangial area; M1, ≥ 4 mesangial cells/mesangial area. (2) Endocapillary hypercellularity (E): E0, absent; E1, present. (3) Segmental glomerulosclerosis (S): S0, absent; S1, present. (4) Tubular atrophy/interstitial fibrosis (T): T0, < 25%; T1, 26%-50%; T2, > 50%. (5) Cellular/fibrocellular crescents (C): C0, absent; C1, crescents present in < 25% of glomeruli; C2, crescents present in ≥ 25% of glomeruli. All pathological diagnoses were made independently by two pathologists.

### Follow-up and study outcomes

In this study, all data from patients were retrospectively collected from the date of IgAN diagnosis to August 2025. All patients were followed up through the outpatient or inpatient systems of the Affiliated Hospital of Chengde Medical University, or via telephone, with a minimum follow-up duration of 12 months. The primary endpoint was defined as progression to ESRD, specifically an eGFR < 15 ml/min/1.73 m² or initiation of renal replacement therapy.

### Statistical analysis

SPSS 26.0 and R 3.4.3 software were used for statistical analysis [17–19]. The normality of measurement data was tested using the Shapiro-Wilk test. Data following a normal distribution were expressed as mean ± standard deviation, and comparisons between groups were performed using the independent samples t-test. Non-normally distributed measurement data were expressed as median (P25, P75) [M (P25, P75)], and comparisons between groups were performed using the Mann-Whitney U test. Categorical data were expressed as frequencies (percentages) [n (%)], and comparisons between groups were performed using the chi-square (χ²) test. The area under the curve (AUC) was calculated using receiver operating characteristic (ROC) curve analysis to evaluate the predictive ability of the NPAR for renal outcomes, and the optimal cut-off value was determined using the Youden index. To systematically evaluate the predictive performance of NPAR compared withsolute neutrophil count×100/albumin), we further conducted a sensitivity analysis.To evaluate the stability of the optimal cut-off value and assess the risk of overfitting, we performed internal validation using 1,000 bootstrap resamples. The optimism-corrected AUC was calculated using the optimism correction method, and the median bootstrap-derived cut-off value with its 95% confidence interval (CI) was reported. Correlation analysis was performed using Spearman’s rank correlation. Kaplan–Meier analysis was used to estimate survival curves.To ensure that the independent effect of the core exposure factor (NPAR) was not excluded by the L1 penalty term, its penalty factor was set to 0 (forced retention) in the LASSO regression, while the remaining variables were subjected to standard L1 penalization for dimensionality reduction. The specific analytical steps included:①candidate variables were preliminarily screened using univariate Cox regression (*P* < 0.1);②LASSO regression with 10-fold cross-validation (using lambda.min as the optimal penalty parameter) was applied to compress the coefficients of irrelevant variables and select key predictors; ③the stability of variable selection was assessed using 1,000 bootstrap resamples, and only variables with a selection rate ≥ 80% were retained; ④before constructing the multivariate Cox regression model (Enter method), the variance inflation factor (VIF) was used to assess collinearity among the included variables, with VIF < 5 as the criterion for no significant multicollinearity; finally, the multivariate Cox regression model was used to calculate hazard ratios (HRs) and their 95% CIs. Variables with *P* < 0.05 were considered independent risk factors for progression to ESRD in patients with IgAN. In addition, subgroup analyses and interaction tests were further performed based on clinical and pathological characteristics to assess the robustness of the results. Restricted cubic splines (RCS) were used to explore the association between the NPAR and poor renal prognosis in patients with IgAN.A *P*-value < 0.05 was considered statistically significant.

## Results

### Baseline characteristics of all study participants

**Fig. 2:**
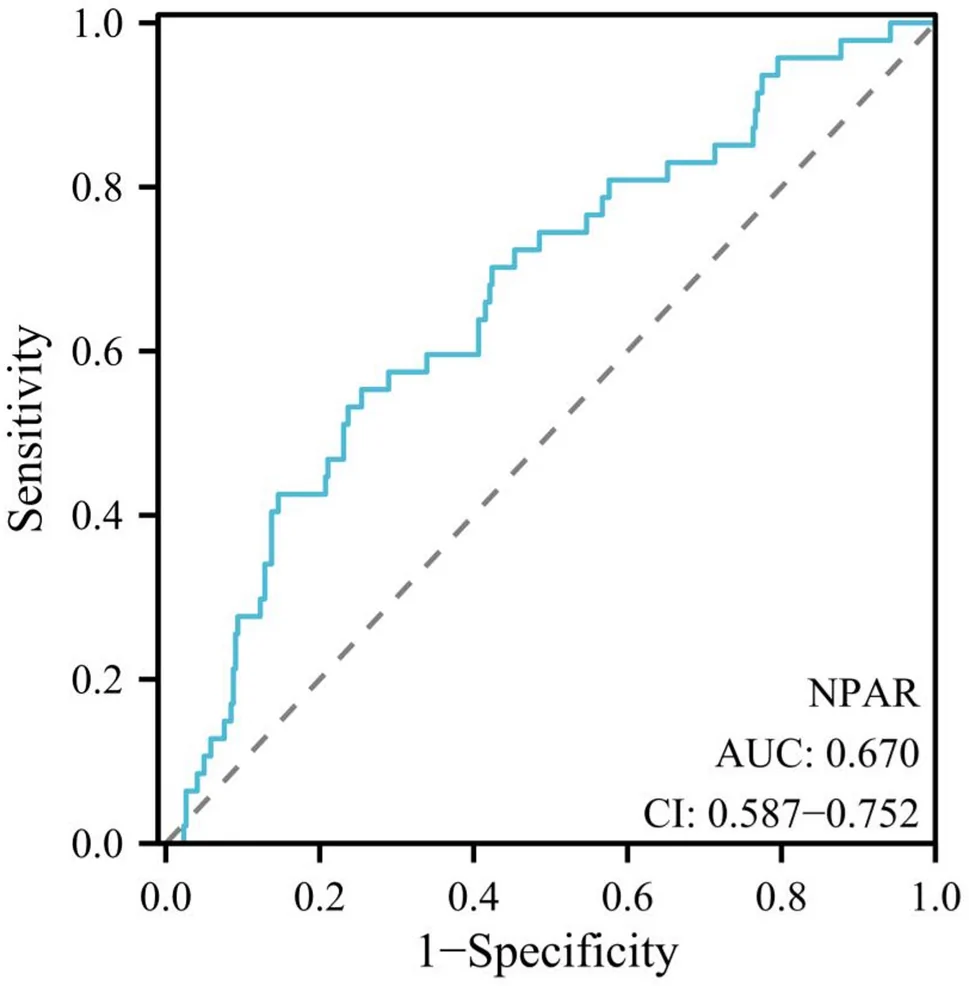
ROC curve of the NPAR for predicting ESRD in IgAN patients

In this study, we used ROC curve analysis to evaluate the predictive value of the NPAR. The results showed that the optimal cut-off value of the NPAR for predicting ESRD was 18.54, with a sensitivity of 55.3% and a specificity of 74.6% (Fig. 2). To further evaluate the stability of this cut-off value and the potential risk of overfitting, we performed internal validation using 1,000 bootstrap resamples. The results showed that the median bootstrap-derived cut-off value was 18.55 (95% CI: 15.48～20.82), which was relatively close to the original Youden index-derived cut-off value (18.54).

After optimism correction, the AUC was 0.668 with an optimism of 0.001, suggesting a relatively low risk of model overfitting and indicating that the AUC estimate had reasonable stability to a certain degree (Supplementary Table S1).

### Sensitivity analysis: NPAR versus aNPAR

Sensitivity analysis showed that NPAR had superior predictive performance for ESRD compared with aNPAR (AUC: 0.670 vs. 0.644; HR: 2.176 vs. 2.007), supporting the rationale for using neutrophil percentage in the definition of NPAR in our study (Supplementary Table S2).

### Baseline characteristics

We ultimately included 389 patients with IgAN in the present study. The median follow-up period for all patients was 41 (24, 74) months, and the median age was 42 (32, 53) years, with men accounting for 51.1%. Based on the optimal cut-off value determined by the ROC curve, patients were divided into a high NPAR group (NPAR ≥ 18.54, *n* = 113) and a low NPAR group (NPAR < 18.54, *n* = 276). Compared to the low NPAR group, patients in the high NPAR group were significantly older and had a significantly higher prevalence of anemia, as well as higher NEUT%, 24-h UPE, and incidence of ESRD (all *P* < 0.05), and significantly lower eGFR and ALB levels. Regarding pathological manifestations, patients in the high NPAR group were more likely to present with E1 (*P* = 0.004) and T1-2 (*P* = 0.023). These findings indicate that patients with high NPAR exhibit more severe clinical and pathological characteristics. In terms of treatment, the proportion of patients receiving corticosteroids or/and immunosuppressive therapy(CS or/and IT) was significantly higher in the high NPAR group compared to the low NPAR group (*P* < 0.001), whereas the proportion receiving angiotensin-converting enzyme inhibitors or angiotensin II receptor blockers(ACEI or ARB) was significantly lower (*P* < 0.001). There were no statistically significant differences between the two groups in terms of sex, prevalence of hypertension, prevalence of hyperuricemia, and PLT (all *P* > 0.05) (Table 1).

**Table 1:** Comparison of baseline demographic, clinical, and pathological data between patients in the low-NPAR and high-NPAR groups

Characteristics	Low NPAR(*n* = 276)	High NPAR(*n* = 113)	*P* value
Age (years)	40(32,51)	47(35,58)	< 0.001
Male	140(50.7%)	59(52.2%)	0.824
Hypertension (%)	158(57.2%)	73(64.6%)	0.211
Hyperuricemia (%)	125(45.2%)	39(34.5%)	0.055
Anemia (%)	20(7.2%)	30(26.5%)	< 0.001
NEUT%(%)	60.7(55.5,65.7)	68.1(63.6,73.1)	< 0.001
PLT(*10^9^/L)	243.5(208.0,288.5)	240.0(201.0,298.0)	0.528
ALB(g/L)	41.1(37.9,43.6)	31.4(27.9,34.6)	< 0.001
24-h UPE (g/d)	2.30(1.28,4.15)	5.77(3.07,10.06)	< 0.001
eGFR(ml/min/1.73m^2^)	81.0(59.2,106.5)	73.0(43.1,101.5)	0.007
M1	230(83.3%)	90(79.6%)	0.384
E1	36(13.0%)	29(25.6%)	0.004
S1	116(42.0%)	45(39.8%)	0.734
T1-2	103(37.3%)	57(50.4%)	0.023
C1-2	67(24.3%)	36(31.8%)	0.130
ACEI or ARB use(%)	225(81.5%)	74(65.4%)	< 0.001
CS or/and IT(%)	61(22.1%)	58(51.3%)	< 0.001
Follow-up (m)	44.5(26.2,74.7)	36.0(20.5,70.5)	0.105
ESRD (%)	21(7.6%)	26(23.0%)	< 0.001

NEUT%: neutrophil percentage (%); PLT: platelet count; ALB: albumin; 24-h UPE: 24-h urine protein excretion; eGFR: estimated glomerular filtration rate; M: mesangial hypercellularity; E: endocapillary hypercellularity; S: segmental glomerulosclerosis; T: tubular atrophy and interstitial fibrosis; C: crescents; ACEI or ARB use: angiotensin-converting enzyme inhibitor or angiotensin II receptor blocker use; CS or/and IT: corticosteroids or/and immunosuppressive therapy; ESRD: end-stage renal disease

### Correlation analysis between the NPAR and clinicopathological parameters

To investigate the relationship between the NPAR and certain clinicopathological parameters, we performed a correlation analysis. The results showed that the NPAR was significantly positively correlated with age (*r* = 0.141, *P* = 0.005), anemia (*r* = 0.270, *P* < 0.001), 24-h UPE (*r* = 0.484, *P* < 0.001), E (*r* = 0.191, *P* < 0.001), and T (*r* = 0.123, *P* = 0.015), and significantly negatively correlated with eGFR (*r* = − 0.155, *P* = 0.002) (Table 2).

**Table 2:** Correlation analysis of the NPAR with clinical and pathological indicators

Indexes	Correlation coefficient	95% CI	*P* value
Age	0.141	0.039～0.240	0.005
Anemia	0.270	0.172～0.362	< 0.001
24-h UPE	0.484	0.401～0.559	< 0.001
eGFR	-0.155	-0.253～-0.053	0.002
E	0.191	0.090～0.287	< 0.001
T	0.123	0.021～0.222	0.015

h UPE: 24-h urine protein excretion; eGFR: estimated glomerular filtration rate; E: endocapillary hypercellularity; T: tubular atrophy and interstitial fibrosis

### The NPAR is an independent risk factor for the progression of IgAN to ESRD

**Fig. 3:**
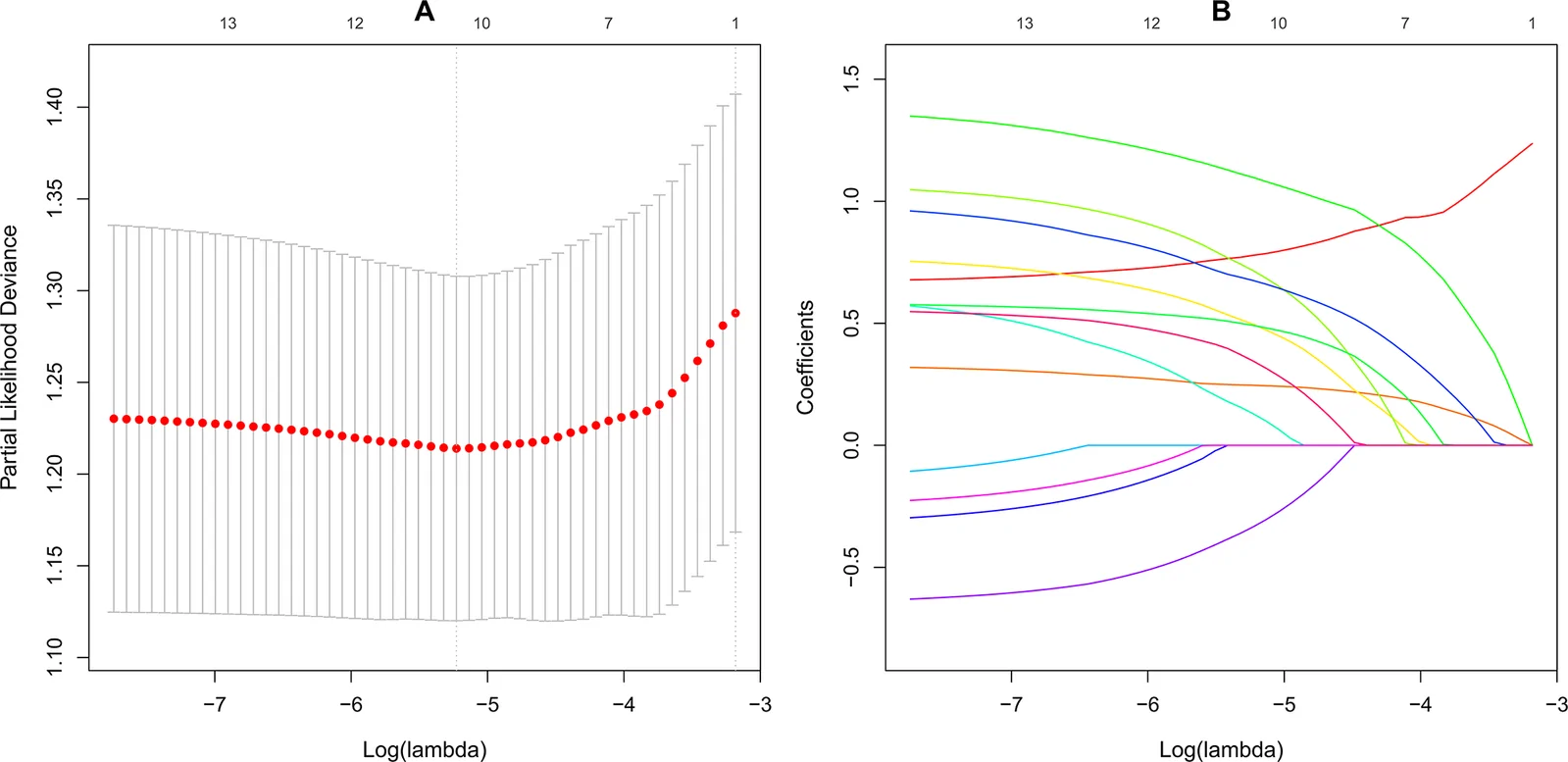
Variable selection by Lasso regression model. **A**: Log Lambda versus partial likelihood deviance. **B**: Log Lambda versus coefficients

In this study, univariate Cox regression analysis was first performed to screen candidate variables using a threshold of *P* < 0.1. The variables identified included high NPAR, male, hypertension, hyperuricemia, anemia, eGFR < 90 ml/min/1.73 m²,24-h UPE, E1, S1, T1-2,C1-2,ACEI/ARB use, and CS or/and IT (Table 3). Subsequently, LASSO-penalized Cox regression was performed for variable dimensionality reduction. To ensure that the independent effect of NPAR was not excluded by the L1 penalty term, its penalty factor was set to 0 (forced retention), while the remaining variables were subjected to standard L1 penalization. Variable selection was conducted using 10-fold cross-validation with lambda.min as the optimal penalty parameter (Fig. 3A, B). The stability of variable selection was further assessed using 1,000 bootstrap resamples. The results showed that, due to the zero penalty factor setting, NPAR was retained in all resampled datasets (selection rate: 100%). Based on the criterion of a bootstrap selection rate ≥ 80%, high NPAR, male, hyperuricemia, anemia, eGFR < 90 ml/min/1.73 m², 24-h UPE, and T1-2 were ultimately included in the multivariate Cox regression model (Table 4). Collinearity diagnostics showed that the variance inflation factor for all included variables was less than 1.5, indicating no significant multicollinearity (Supplementary Table S3). The three selected variables were then entered into a multivariate Cox regression model. The results demonstrated that high NPAR (≥ 18.54) was an independent risk factor for progression to ESRD in patients with IgAN (HR = 2.176, 95%CI: 1.058～4.472, *P* = 0.034) (Table 4).

**Table 3:** Univariate Cox regression analyses of renal survival in patients with IgAN

Characters	Univariate cox regression	
	HR(95% CI)	*P* value
High NPAR	3.443(1.935～6.124)	< 0.001
Age	0.990(0.966～1.014)	0.402
Male	1.915(1.062～3.451)	0.031
Hypertension	2.112(1.114～4.003)	0.022
Hyperuricemia	1.938(1.089～3.450)	0.024
Anemia	5.025(2.628～9.607)	< 0.001
PLT	0.998(0.993～1.003)	0.455
eGFR<90 ml/min/1.73m^2^	10.618(3.802～29.654)	< 0.001
24-h UPE	6.626(0.914～48.055)	0.061
M1/M0	1.181(0.584～2.386)	0.644
E1/E0	2.940(1.385～6.242)	0.005
S1/S0	1.879(1.058～3.335)	0.031
T1-2/T0	5.418(2.757～10.647)	< 0.001
C1-2/C0	1.694(0.924～3.106)	0.088
ACEI or ARB use	0.460(0.256～0.824)	0.009
CS or/and IT	2.105(1.179～3.759)	0.012

NPAR: neutrophil percentage-to-albumin ratio; PLT: platelet count; eGFR: estimated glomerular filtration rate; 24-h UPE: 24-h urine protein excretion; M: mesangial hypercellularity; E: endocapillary hypercellularity; S: segmental glomerulosclerosis; T: tubular atrophy and interstitial fibrosis; C: crescents; ACEI or ARB use: angiotensin-converting enzyme inhibitor or angiotensin II receptor blocker use; CS or/and IT: corticosteroids or/and immunosuppressive therapy

**Table 4:** LASSO regression and multivariate Cox regression analysis of risk factors for adverse renal prognosis in IgAN

Characters	Lasso coefficients	Bootstrap selection rate (%)	Multivariate cox regression	
			HR(95% CI)	*P* value
High NPAR	0.7786	100	2.176(1.058～4.472)	0.034
Male	0.4957	92.8	1.818(0.985～3.356)	0.056
Hyperuricemia	0.4916	89.0	2.177(1.130～4.196)	0.020
Anemia	0.7159	89.6	2.399(1.186～4.851)	0.015
eGFR<90 ml/min/1.73m^2^	1.0978	99.4	4.681(1.567～13.990)	0.006
24-h UPE	0.2459	95.6	1.075(1.001～1.155)	0.048
T1-2/T0	0.6754	97.2	2.406(1.172～4.938)	0.017

NPAR: neutrophil percentage-to-albumin ratio; eGFR: estimated glomerular filtration rate; 24-h UPE: 24-h urine protein excretion; T: tubular atrophy and interstitial fibrosis

#### Prognostic value of NPAR in patients with IgAN: subgroup analyses based on clinical and pathological characteristics

**Fig. 4:**
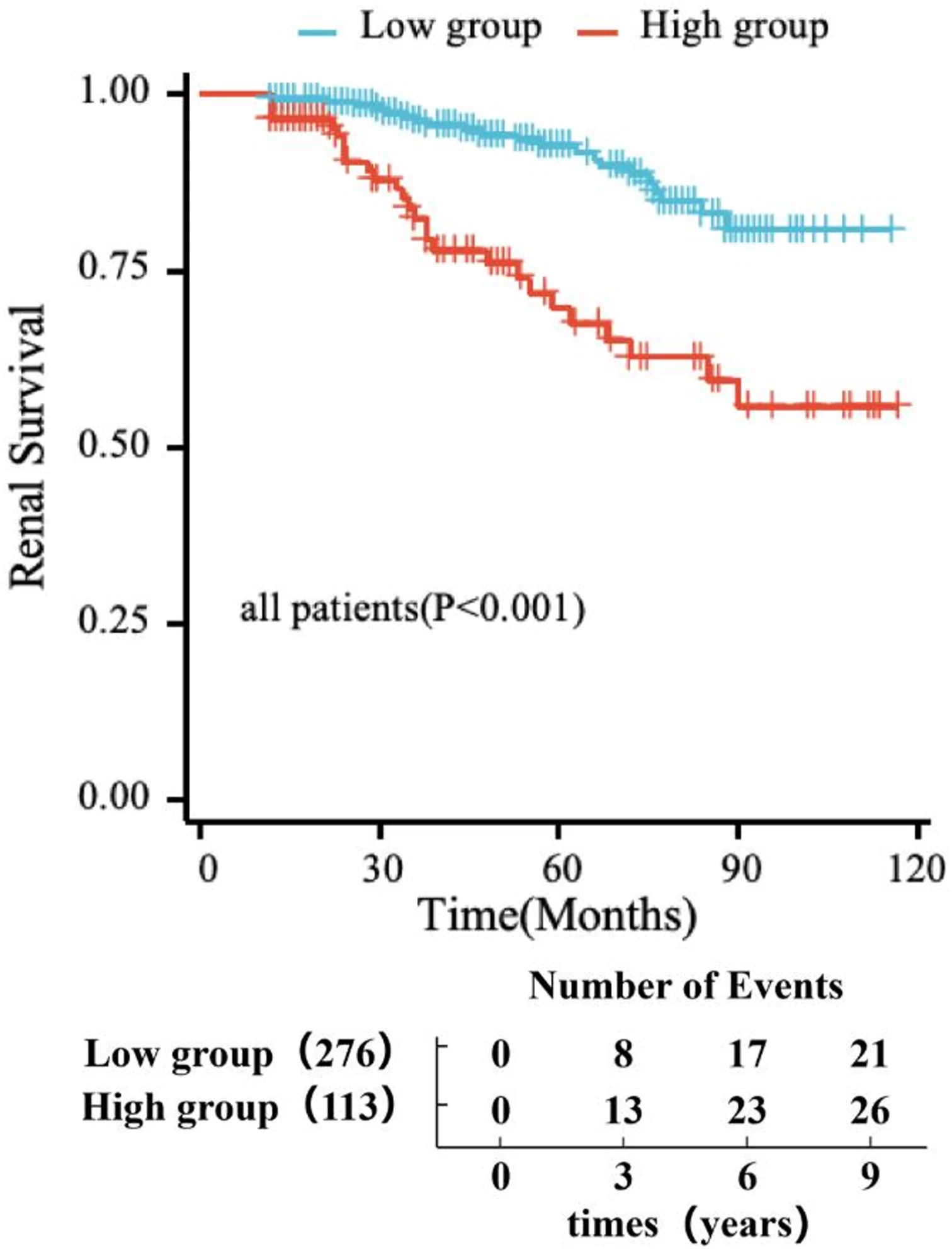
Kaplan–Meier analysis for the endpoint of ESRD stratified according to the cutoff point of NPAR. The cumulative counts of endpoint events for each group at 0, 3, 6, and 9 years are shown in the figure, respectively

**Fig. 5:**
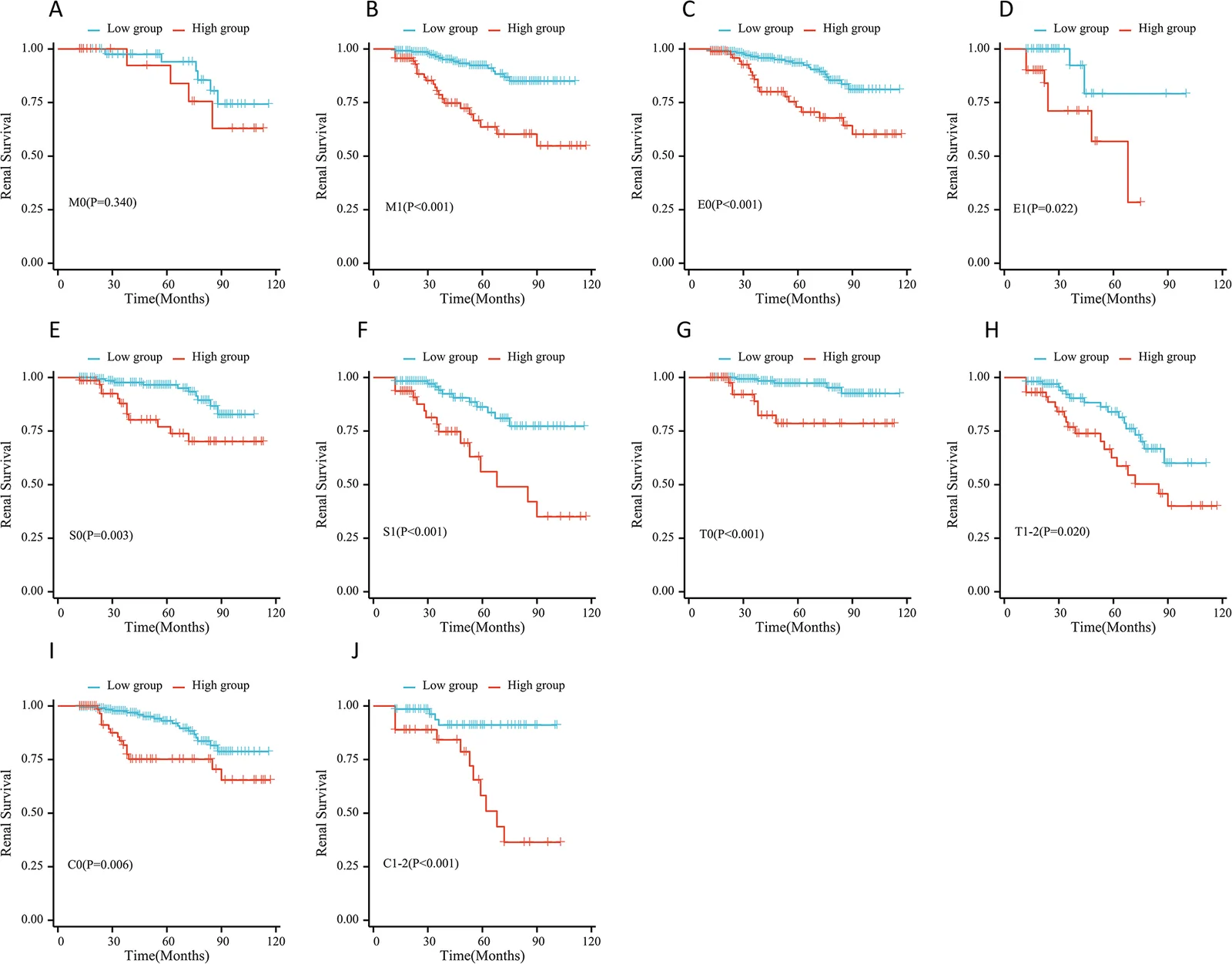
Subgroup analysis of patients with IgAN with different types of pathological lesions. Kaplan–Meier curve of patients with M0 (**A**), M1 (**B**), E0 (**C**), E1 (**D**), S0 (**E**), S1 (**F**), T0 (**G**), T1-2 (**H**), C0 (**I**), C1-2 (**J**) in two groups stratified by NPAR

**Fig. 6:**
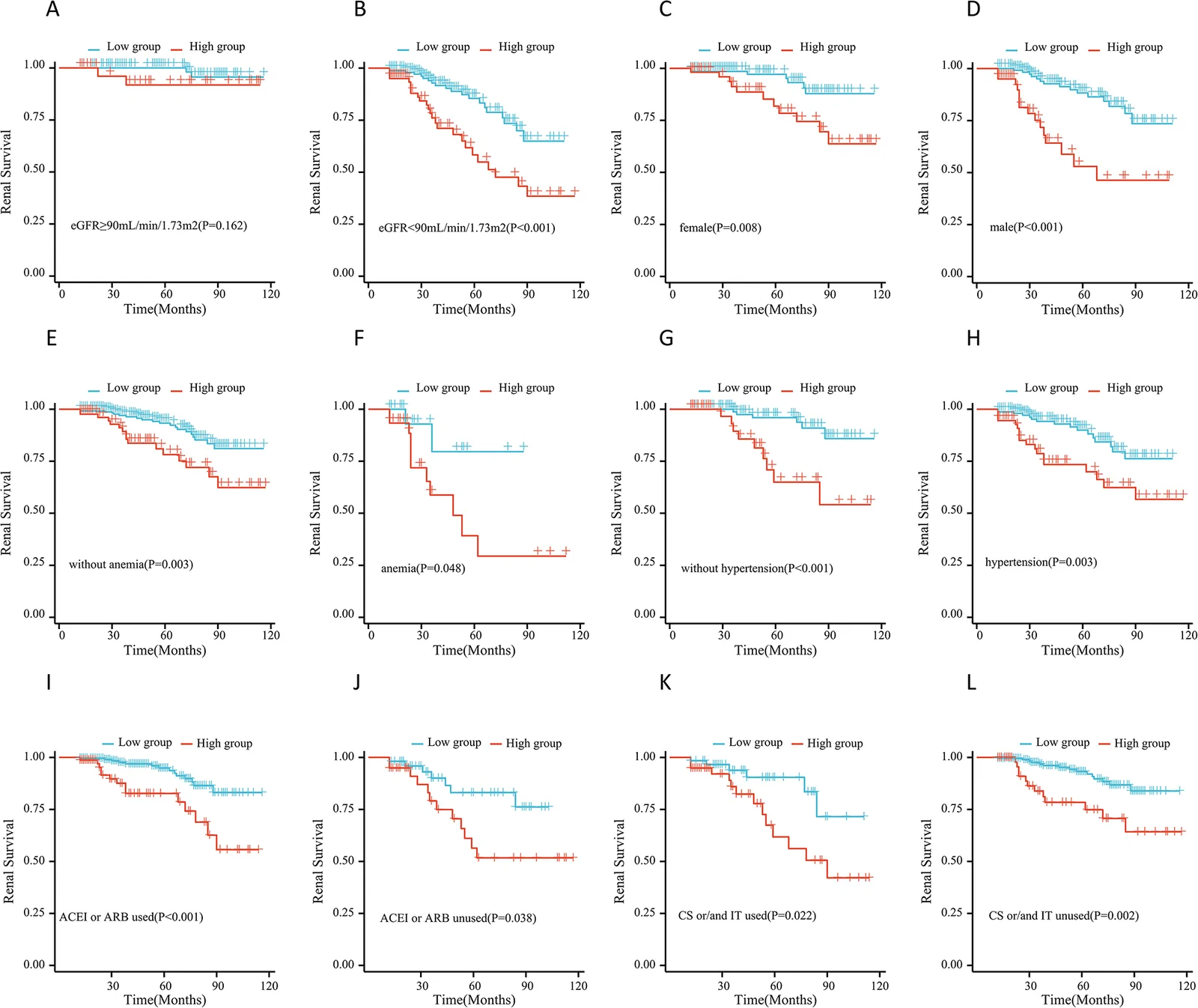
Subgroup analysis of patients with IgAN with different clinical characteristics. Kaplan–Meier curve of patients with eGFR ≥ 90 ml/min/1.73 m² (**A**), eGFR < 90 ml/ min/1.73 m² (**B**), female (**C**), male (**D**), normal hemoglobin (**E**), anemia (**F**), normotension (**G**), hypertension (**H**), ACEI or ARB used (**I**), ACEI or ARB unused (**J**), CS or/and IT used (**K**), and CS or/and IT unused (**L**) in two groups distinguished by NPAR

Kaplan–Meier survival curves revealed that high NPAR (≥ 18.54) was significantly associated with poorer renal survival in patients with IgAN (*P* < 0.001), accompanied by a higher cumulative incidence of renal endpoint events (Fig. 4). Subsequently, we performed subgroup analyses based on different clinical manifestations (Fig. 5) and pathological features (Fig. 6). The results showed that NPAR demonstrated a certain prognostic discriminative trend in the following pathological subtypes: M1 (*P* < 0.001, Fig. 5B), E0 (*P* < 0.001, Fig. 5C), E1 (*P* = 0.022, Fig. 5D), S0 (*P* = 0.003, Fig. 5E), S1 (*P* < 0.001, Fig. 5F), T0 (*P* < 0.001, Fig. 5G), T1-2 (*P* = 0.020, Fig. 5H), C0 (*P* = 0.006, Fig. 5I), and C1-2 (*P* < 0.001, Fig. 5J). However, no similar trend was observed in patients with the M0 subtype. A post-hoc power analysis was performed for the M0 subgroup. This subgroup consisted of 69 patients with only 10 ESRD events, and the ratio of high to low NPAR groups was approximately 1:2. Under a two-sided α level of 0.05, the statistical power to detect an HR of 2.0 was only 17.8%.In addition, NPAR showed a certain prognostic discriminative tendency in patients with reduced renal function, different sexes, varying hemoglobin levels, and different blood pressure statuses (Figs. 6B-H), and this trend was not markedly influenced by ACEI/ARB (Figs. 6I-J) or CS or/and IT treatment (Figs. 6K-L).

### Non-linear association between the NPAR and poor prognosis in patients with IgAN

**Fig. 7:**
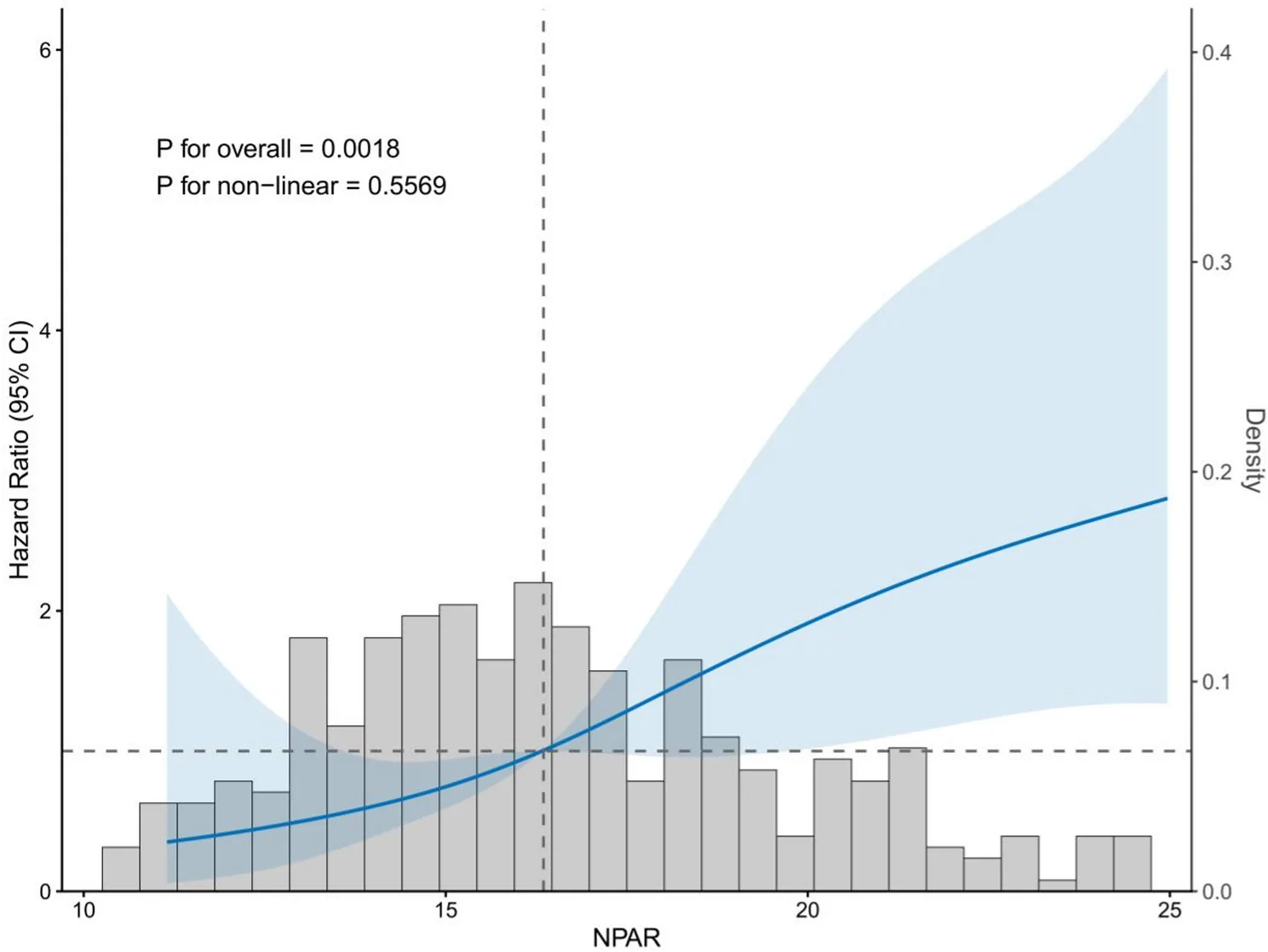
Restricted cubic spline showing the relationship between the NPAR and adverse renal prognosis in patients with IgAN.The solid blue line represents the hazard ratio (HR), and the light blue shaded area indicates the 95% CI. The gray histogram shows the frequency distribution of NPAR in the study population, with the right vertical axis corresponding to the kernel density of NPAR. The *P* values for the overall association (*P* for overall) and for non-linearity (*P* for non-linearity) are indicated in the figure

RCS analysis was further employed to explore the association between NPAR and poor renal prognosis in patients with IgAN. The results showed that the overall association between NPAR and poor renal prognosis was statistically significant (*P* for overall = 0.0018). The test for non-linearity indicated a linear positive correlation between NPAR and poor renal prognosis (*P* for non-linearity = 0.5569), indicating that higher NPAR levels were associated with a corresponding increase in the risk of progression to ESRD (Fig. 7).

## Discussion

IgAN is a complex kidney disease resulting from the interplay between genetic predisposition and environmental factors. Although its exact pathogenesis has not been fully elucidated, evidence indicates that immune dysregulation and inflammatory responses play a central role in the initiation and progression of the disease [20]. Therefore, further investigation into the inflammatory mechanisms associated with IgAN is conducive to a deeper understanding of its pathophysiological processes and provides an important theoretical basis for the early identification of high-risk patients and the development of novel interventional strategies.

In recent years, the NPAR, a novel composite indicator integrating inflammatory response and nutritional status, has garnered widespread attention. Previous studies have confirmed that IgAN is characterized by the aberrant production of galactose-deficient IgA1 (Gd-IgA1). When Gd-IgA1 levels are elevated, the immune complexes formed deposit in the glomerular mesangial area, which can stimulate mesangial cell proliferation and induce the local production of various cytokines and chemokines, such as IL-6 and TGF-β [21]. These factors, in turn, activate neutrophils, promoting the release of reactive oxygen species, inflammatory mediators, and neutrophil extracellular traps. These substances can induce damage to the glycocalyx layer and affect cell surface adhesion molecules. Additionally, receptor-mediated vascular reactivity, endothelial barrier function, and activation of the coagulation system are also impaired. These inflammation-induced alterations ultimately lead to microvascular damage, glomerular lesions, and tubulointerstitial fibrosis [22, 23].

ALB, the most abundant protein in plasma (accounting for approximately 50%–60%) [24], contains numerous cysteine residues in its molecular structure, which endows it with significant antioxidant and anti-inflammatory capabilities [25, 26]. Under physiological conditions, ALB can effectively neutralize the reactive oxygen species produced by inflammatory cells, protecting renal cells from oxidative damage. However, in the state of hypoalbuminemia, this protective mechanism is significantly weakened. This exacerbates inflammation and oxidative stress mediated by factors such as IL-6 and TNF-α and impairs the body’s ability to clear oxygen free radicals [27]. Furthermore, the inflammatory response itself can further inhibit ALB synthesis and promote its consumption. Previous studies have shown that inflammatory cytokines can suppress the synthesis of albumin and its mRNA in the liver [28], whereas factors such as interleukin-2 can cause abnormal albumin metabolism. Oxidative stress can also lead to structural denaturation of ALB, thus forming a vicious cycle that continuously aggravates the body’s inflammatory and injury state [29]. Previous studies have confirmed that low serum ALB is an independent risk factor for the progression of IgAN to end-stage renal disease, with every 1 g/dL decrease in ALB increasing the risk of end-stage renal disease 1.89 times [30]. In oxidative stress models of mesangial cells and human renal cells induced by hydrogen peroxide, ALB inhibited intracellular reactive oxygen species production and mitochondrial damage in a dose-dependent manner, further highlighting its critical role in renal protection [31]. The NPAR integrates both the neutrophil percentage and ALB concentration, providing a more comprehensive assessment. It can more accurately represent the inflammatory state, suggesting that a high NPAR may influence the progression of IgAN by promoting inflammation, inducing cytokine release, and exacerbating oxidative stress.It should be noted that the above-mentioned potential mechanisms of NPAR are primarily based on theoretical inferences from the existing literature. As this study is a retrospective clinical cohort study, we did not measure inflammatory markers such as IL-6, TNF-α, or CRP in the peripheral blood of the participants, nor did we assess the redox status of albumin. Therefore, the above pathways remain within the realm of hypothesis, and the exact molecular mechanisms warrant further validation through subsequent basic and clinical studies.

To validate the relevance of the aforementioned theoretical mechanisms in a clinical cohort, we retrospectively analyzed 389 patients with biopsy-proven IgAN. The predictive value of the NPAR for poor renal prognosis in IgAN was evaluated using ROC curve analysis, which showed an area under the curve of 0.670 for predicting adverse renal outcomes. Patients were divided into high and low NPAR groups based on the optimal cut-off value (18.54). Further analysis indicated that an elevated NPAR was significantly associated with more severe clinicopathological phenotypes in patients with IgAN. Regarding clinical indicators, the NPAR was positively correlated with 24-h UPE, consistent with previous research findings. A cross-sectional study involving 2,263 American adults with type 2 diabetes mellitus also found a positive correlation between the NPAR and proteinuria [32]. The NPAR was negatively correlated with eGFR, also aligning with prior studies. Previous research has demonstrated a negative correlation between eGFR and neutrophil count, and a positive correlation between eGFR and ALB levels [33, 34]. This study found a positive correlation between the NPAR and anemia, suggesting that the chronic inflammatory state reflected by an elevated NPAR may inhibit hematopoietic function through multiple pathways. On one hand, the accumulation of uremic toxins accompanying the decline in renal function interferes with the synthesis and action of erythropoietin (EPO) [35]; on the other hand, inflammatory factors such as TNF-α can directly inhibit the proliferation and differentiation of erythroid progenitor cells and reduce their responsiveness to EPO, collectively hindering erythropoiesis [36]. In terms of pathological damage, patients in the high NPAR group were more prone to severe lesions, including endocapillary hypercellularity, tubular atrophy/interstitial fibrosis, and cellular crescent formation. The findings of this study address a gap in knowledge regarding the relationship between the NPAR and these specific renal pathological injuries in patients with IgAN.

In the prognostic analysis, Kaplan–Meier survival analysis revealed that the cumulative renal survival rate was significantly lower in the high NPAR group than in the low NPAR group. Multivariate Cox regression analysis further showed that elevated NPAR was an independent risk factor for progression to ESRD in patients with IgAN. RCS analysis also suggested a significant linear positive correlation between NPAR levels and the risk of ESRD. To more comprehensively evaluate the prognostic value of NPAR in patients with different clinicopathological characteristics, we performed subgroup analyses. When stratified according to the Oxford classification criteria, NPAR showed a certain prognostic discriminative trend in the M1, E0, E1, S0, S1, T0, T1-2, C0, and C1-2 subgroups, whereas no similar trend was observed in the M0 subgroup.This difference may stem from the disparity in disease activity represented by M1 versus M0. M1 indicates significant mesangial hypercellularity, often accompanied by active inflammatory responses. Consequently, in such patients, the NPAR, an indicator reflecting systemic inflammatory status, is naturally closely related to disease activity and prognosis. Conversely, M0 represents mild pathological changes, where clinical progression may be more driven by non-inflammatory factors (such as hemodynamic abnormalities or fibrotic processes), thereby diminishing the prognostic discriminative ability of NPAR in this subgroup. In addition to these pathological factors, the post-hoc power analysis presented in the Results section indicated that the M0 subgroup had a limited sample size and a small number of events (power of only 17.8%), suggesting that insufficient statistical power may also have contributed to the lack of a significant predictive effect of NPAR in this subgroup.In addition, NPAR showed a certain prognostic discriminative tendency in patients with reduced renal function, with or without anemia, with different blood pressure statuses (normal or elevated), across sexes, and regardless of whether they received ACEI/ARB or CS/IT treatment. These results seem to suggest that its prognostic value might be higher in patients with severe disease. The underlying reason for this phenomenon may be the difference in the rate of disease progression: compared to patients with mild, slowly progressing disease, renal function deteriorates rapidly in severely ill patients who reach endpoints such as ESRD more quickly. This provides a clearer predictive window and discriminatory space for the NPAR [37]. However, this study only identified a modest but statistically significant association between NPAR and poor renal prognosis in patients with IgAN (AUC = 0.670). This finding warrants further investigation but is insufficient for clinical implementation at this stage and requires external validation.

In this study, baseline treatment strategies differed significantly between the two groups. Patients in the high NPAR group had more severe disease and were more likely to receive CS or/and IT, whereas those in the low NPAR group, with milder disease, had a higher rate of ACEI/ARB use. This pattern reflects real-world clinical practice. Both classes of drugs exert renoprotective effects, which may introduce reverse confounding in the observed disease trajectory and outcomes. Specifically, CS or/and IT may delay renal function decline in the high NPAR group, potentially leading to an underestimation of the adverse prognostic effect of NPAR. Conversely, the higher use of ACEI/ARB in the low NPAR group provides additional renal protection. Despite this reverse confounding, subgroup analyses showed that the prognostic performance of NPAR was relatively consistent across different treatment statuses, supporting its potential as an independent prognostic marker to some extent.Notably, the primary objective of this study was to evaluate the prognostic value of baseline NPAR, rather than to assess the therapeutic effects of these medications. Although subgroup analyses were performed to explore the influence of treatment factors on the results, the retrospective design of this study limits our ability to completely exclude potential interference from indication bias in treatment allocation.

However, this study has some limitations. First, it is a single-center, retrospective study with a limited sample size. The participants were enrolled from a single hospital, and the majority were patients with IgAN from northern China, which may limit the generalizability of the results. Future multi-center, prospective studies are needed to further validate the universal applicability of the NPAR in IgAN. Second, owing to the constraints of the study design, we were unable to investigate the impact of long-term dynamic changes in NPAR levels on the renal prognosis of IgAN. Additionally, we could not account for the influence of a small number of potential confounding factors (e.g., patients’ dietary habits, presence of chronic inflammation) on the results. Third, the M0 subgroup had a small sample size and limited endpoint events. Post-hoc power analysis showed a statistical power of only 17.8%, and NPAR failed to show a predictive effect in this subgroup. Therefore, relevant conclusions need further verification in larger samples.Fourth, the AUC of NPAR for predicting ESRD in this study was 0.670, indicating its limited predictive ability as a standalone marker and making it insufficient to guide clinical decisions independently. Furthermore, the cut-off value was derived from single-center data, and its applicability across different populations remains to be externally validated. Therefore, the use of NPAR as a single indicator in clinical practice should be approached with caution, and it is recommended to combine it with other clinical and pathological parameters for a more comprehensive assessment of patient prognosis. Future studies aim to expand the sample size and conduct multi-center external validation to further improve the model’s robustness and clinical generalizability. Fifth, the proposed mechanism by which NPAR may influence the progression of IgAN lacks direct evidence from peripheral blood inflammatory markers or assessment of albumin redox status. The exact molecular mechanisms remain to be further validated through subsequent basic and clinical studies.In addition, this study has a retrospective design, and treatment allocation was subject to indication bias. Due to incomplete documentation of medication adjustments during follow-up and the role of treatment as a mediator, treatment was not included as a time-varying covariate in the Cox regression model. Although subgroup analyses were performed to explore the influence of treatment factors on the results, the retrospective design of this study limits our ability to completely exclude potential interference from indication bias in treatment allocation.Future prospective studies will systematically document dynamic medication changes and incorporate them as a time-dependent covariate to optimize the model and enhance the robustness of the conclusions.

## Conclusion

In conclusion, elevated NPAR, as a convenient and cost-effective inflammatory marker, was independently associated with more severe clinicopathological phenotypes and adverse renal outcomes in patients with IgAN. Nevertheless, the observed association was modest (AUC = 0.670) and should be considered a hypothesis-generating exploratory finding that is not yet ready for clinical application. External validation is warranted to confirm this finding.

## Electronic supplementary material

Below is the link to the electronic supplementary material.

Supplementary Material 1

## Data Availability

The datasets used and/or analyzed during the current study are available from the corresponding author on reasonable request.
