# Supplementary material for: Prognostic value of the neutrophil percentage-to-albumin ratio in patients with IgA nephropathy
Source: BMC Nephrol. 2026 Jul 29;27:632. doi: 10.1186/s12882-026-05235-y (PMC13711761; doi:10.1186/s12882-026-05235-y)
Supplement: Supplementary file 1 [file 12882_2026_5235_MOESM1_ESM.docx]

Supplementary Table S1. Bootstrap validation results of NPAR for predicting ESRD

| Parameter | Original value | Bootstrap-estimated value (95% CI) |
| --- | --- | --- |
| Optimal cut-off value | 18.54 | 18.55 (15.48～20.82) |
| AUC | 0.670 | 0.668 (optimism-corrected) |
| Optimism | — | 0.001 |

Supplementary Table 2.Sensitivity analysis: NPAR versus aNPAR

| Characters | AUC（95% CI） | Multivariate cox regression | |
| --- | --- | --- | --- |
|  |  | HR(95% CI) | *P* value |
| NPAR | 0.670（0.587～0.752） | 2.176（1.058～4.472） | 0.034 |
| aNPAR | 0.644（0.551～0.738） | 2.007（1.057～3.807） | 0.033 |

| Characters | VIF |
| --- | --- |
| High NPAR | 1.323 |
| Male | 1.027 |
| Hyperuricemia | 1.067 |
| Anemia | 1.130 |
| eGFR<90ml/min/1.73m^2^ | 1.288 |
| 24-h UPE | 1.282 |
| T1-2/T0 | 1.235 |

Supplementary Table S3. Collinearity diagnostics of variables included in the multivariate Cox regression model
